# Reiki and related therapies in the dialysis ward: an evidence-based and ethical discussion to debate if these complementary and alternative medicines are welcomed or banned

**DOI:** 10.1186/1471-2369-14-129

**Published:** 2013-06-21

**Authors:** Martina Ferraresi, Roberta Clari, Irene Moro, Elena Banino, Enrico Boero, Alessandro Crosio, Romina Dayne, Lorenzo Rosset, Andrea Scarpa, Enrica Serra, Alessandra Surace, Alessio Testore, Nicoletta Colombi, Barbara Giorgina Piccoli

**Affiliations:** 1 SS Nephrology ASOU, san Luigi (regione Gonzole 10), Orbassano 10043, Torino, Italy; 2 Medical School, Università degli Studi di Torino, san Luigi, (regione Gonzole 10), Orbassano 10043, Torino, Italy; 3 Biomedical Library Università degli Studi di Torino, san Luigi, (regione Gonzole 10), Orbassano 10043, Torino, Italy

**Keywords:** Chronic pain, Chronic kidney disease, Alternative, Allied and complementary medicine, Reiki, Touch therapy

## Abstract

**Background:**

Complementary and Alternative Medicines (CAMs) are increasingly practiced in the general population; it is estimated that over 30% of patients with chronic diseases use CAMs on a regular basis. CAMs are also used in hospital settings, suggesting a growing interest in individualized therapies. One potential field of interest is pain, frequently reported by dialysis patients, and seldom sufficiently relieved by mainstream therapies. Gentle-touch therapies and Reiki (an energy based touch therapy) are widely used in the western population as pain relievers.

By integrating evidence based approaches and providing ethical discussion, this debate discusses the pros and cons of CAMs in the dialysis ward, and whether such approaches should be welcomed or banned.

**Discussion:**

In spite of the wide use of CAMs in the general population, few studies deal with the pros and cons of an integration of mainstream medicine and CAMs in dialysis patients; one paper only regarded the use of Reiki and related practices. Widening the search to chronic pain, Reiki and related practices, 419 articles were found on Medline and 6 were selected (1 Cochrane review and 5 RCTs updating the Cochrane review). According to the EBM approach, Reiki allows a statistically significant but very low-grade pain reduction without specific side effects. Gentle-touch therapy and Reiki are thus good examples of approaches in which controversial efficacy has to be balanced against no known side effect, frequent free availability (volunteer non-profit associations) and easy integration with any other pharmacological or non pharmacological therapy. While a classical evidence-based approach, showing low-grade efficacy, is likely to lead to a negative attitude towards the use of Reiki in the dialysis ward, the ethical discussion, analyzing beneficium (efficacy) together with non maleficium (side effects), justice (cost, availability and integration with mainstream therapies) and autonomy (patients’ choice) is likely to lead to a permissive-positive attitude.

**Summary:**

This paper debates the current evidence on Reiki and related techniques as pain-relievers in an ethical framework, and suggests that physicians may wish to consider efficacy but also side effects, contextualization (availability and costs) and patient’s requests, according also to the suggestions of the Society for Integrative Oncology (tolerate, control efficacy and side effects).

## Background

Complementary or allied-alternative medicines (CAMs) are increasingly being used, in particular in patients affected by chronic diseases or diseases “without therapy” [1-3]. The world prevalence of CAMs varies considerably (35-75% in non-selected general populations); in this context, the reluctance to admit CAM use may underestimate it [4-15]. On the other hand, the inclusion of prayer, which is usually considered as a part of the CAMs, can double their prevalence; this is an interesting and highly discussed issue, as not all Authors agree to consider religious beliefs as a part of a therapeutic pathway. However, for the sake of the present review, we would like to mention that one of the first randomized controlled trials on CAMs published on a core clinical journal, the MANTRA trial, regarded the healing effect of prayer [12].

A few reports have dealt with the use of CAMs in Nephrology and Dialysis, underlining their growing diffusion and the need for specific education in renal medicine [16,17].

The opening of the “conventional” to the “complementary” raises new problems: the rapid increase in demand for CAMs requires an adequate medical education and a change in the attitude of hospitals and physicians towards CAMs. According to a 2001 survey, CAMs were taught in about 40% of European medical schools and in 64% of USA ones [18-20]. While several problems remain to be solved (primarily the lack of certification and controls), the position statements of some leading medical societies highlight the responsibility of medical doctors to counsel and guide patients along this complex pathway [21]. Resolution No. 400, May 1997 of the European Parliament and Resolution No. 1206, November 1999 of the Council of Europe stress the need to guarantee citizens the greatest freedom of choice of treatment, ensuring the highest level of security and the most accurate information on the safety, quality and effectiveness of non-conventional treatments, inviting member states to provide information on CAMs [22].

The National Institutes of Health of the USA has a dedicated centre and a site (National Center for Complementary and Alternative Medicine, NCCAM). CAMs are also acquiring space in the Cochrane Collaboration and some important series, such as the BMC, have dedicated a journal to CAMs dealing with studies supported by the National Institutes of Health [23-25]. The Qualitative Methods Working Group of NIH developed a methodological manifesto in 1997 to identify study designs and analyses applicable to CAMs, pursuing standardization or suggesting new approaches, such as the “Whole System Approach”, aimed at respecting the personalization of therapies, which is often basic to the practice of CAMs [24-33]. Within these limits, the application of evidence-based medicine (EBM) to the analysis of CAMs confirms the versatility of EBM as a problem-solving approach, disentangling the complex relationship between “Medicine and Medicines” [34-37].

The discussion of the case of Reiki may highlights the controversial points in the discussion on the attitude towards CAMs in the dialysis ward, and the problem-solving approach integrating EBM with a formal ethical outline, developed in the context of the EBM course of the san Luigi Medical School, may represent an example applicable on other CAMs in similar settings [38-42].

## Discussion

### The interest for Reiki and related CAMs is high in the western population, but the “usual” sources of information are limited and often of low quality

The increasing interest in “non-conventional” approaches is a leading theme in our society [1-3,43-46]. In this context, the so-called “mind and body therapies”, healing touch or Reiki, may represent a prototype of non-medical approaches in a highly “medicalized” population such as dialysis patients.

Reiki (霊気) is a Japanese word meaning “universal life energy”; it is a healing practice consisting in the light laying of hands on or just above the person, with the theoretical goal of facilitating the person’s healing response by getting in touch with the universal energy, which is thought to support the body’s innate capacity for self-healing [47]. Reiki can also be practiced as self-treatment (self-help) [48-51].

Reiki was described in detail by the Japanese master Dr. Mikao Usui in the early 1900s through his study of ancient Tibetan healing arts and the laying on of hands healing tradition. It was brought to the mainland United States via Hawaii during the 1940s, and was introduced into Europe in the 1980s. Treatment consists in at least four sessions of 30–90 minutes, in which the practitioner places his/her hands lightly on or just above the client’s body, palms down, using different hand positions [47].

The popularity of Reiki is increasing in several countries, probably because the healing approach is non-traumatic and easily integrated with conventional therapies [52,53]. In spite of its diffusion, the baseline mechanism of action has not been demonstrated, as the few attempts to investigate it have led to inconsistent results [54].

For the sake of the present analysis, the evidence was retrieved by two pathways, mimicking the patient’s and the physician’s side.

The first search (patient’s perspective) was performed as a tool to define “what the patient knows” as basis for an evidence-based, informed discussion. A non-systematic search on Google and Yahoo, increasingly used both as a tool to better understand patients’ requests and as a clinical problem-solving strategy, confirms the interest in the subject. The large number of citations retrieved with the single term “Reiki” on the most common search engines, plus over 1000 relevant titles on Medline, provided preliminary contextualization and support of the patient’s request. However, the high number of commercial sites on Google and Yahoo should be a warning about the economic pressure (Table 1) [55-61].

**Table 1:** Evidence retrieved on web search engines: quantitative analysis of the first 2 pages of Google and Yahoo

**Search terms**	**Search engine**	**Items**	**Commercial/non-commercialsites in the first 2 pages**	**Sites providing references (non-commercial links in the first 2 pages)**	**No. sites in common in Google and Yahoo**
Reiki	Google	58500000	6/14	3	6
	Yahoo	44900000	6/14	6	
Reiki medicine	Google	17800000	17/3	2	1
	Yahoo	5690000	19/1	1	
Reiki Torino	Google	3680000	18/2	0	0
	Yahoo	51100	10/10	0	
Reiki use	Google	3060000	18/2	2	0
	Yahoo	55300	20/0	0	
Reiki pain	Google	10700000	1/19	9	4
	Yahoo	7150000	2/18	4	
Reiki dialysis	Google	590000	4/16	0	7
	Yahoo	193000	6/14	0	
Reiki cost effectiveness	Google	333000	12/8	12	5
	Yahoo	112000	15/5	9	
Reiki contra- indications	Google	120000	9/11	2	9
	Yahoo	30900	6/14	1	

Legend: * number of links retrieved in first 2 pages in Google and Yahoo.

### The classic EBM approach, based upon treatment efficacy, underlines the limited evidence on Reiki and related CAMs and the low-grade effect on pain

This conclusion stems from a second search (physician’s perspective), that was performed on Pubmed and CINAHL, according with the classic rules of EBM database searches.

Dialysis is a very specific niche for complex heterogeneous patients, often with high comorbidity; it is rare to find efficacy studies on CAMs tailored to this population. In fact, during a first, preliminary search analysis combining the free terms “Reiki”, “Dialysis” and “Pain”, very few papers were retrieved (8 papers matching “Reiki” and “Dialysis”, 3 also with “Pain”), but only one paper dealt with such a case, leading us to broaden the search strategy to “Reiki and pain” [62].

**Figure 1:**
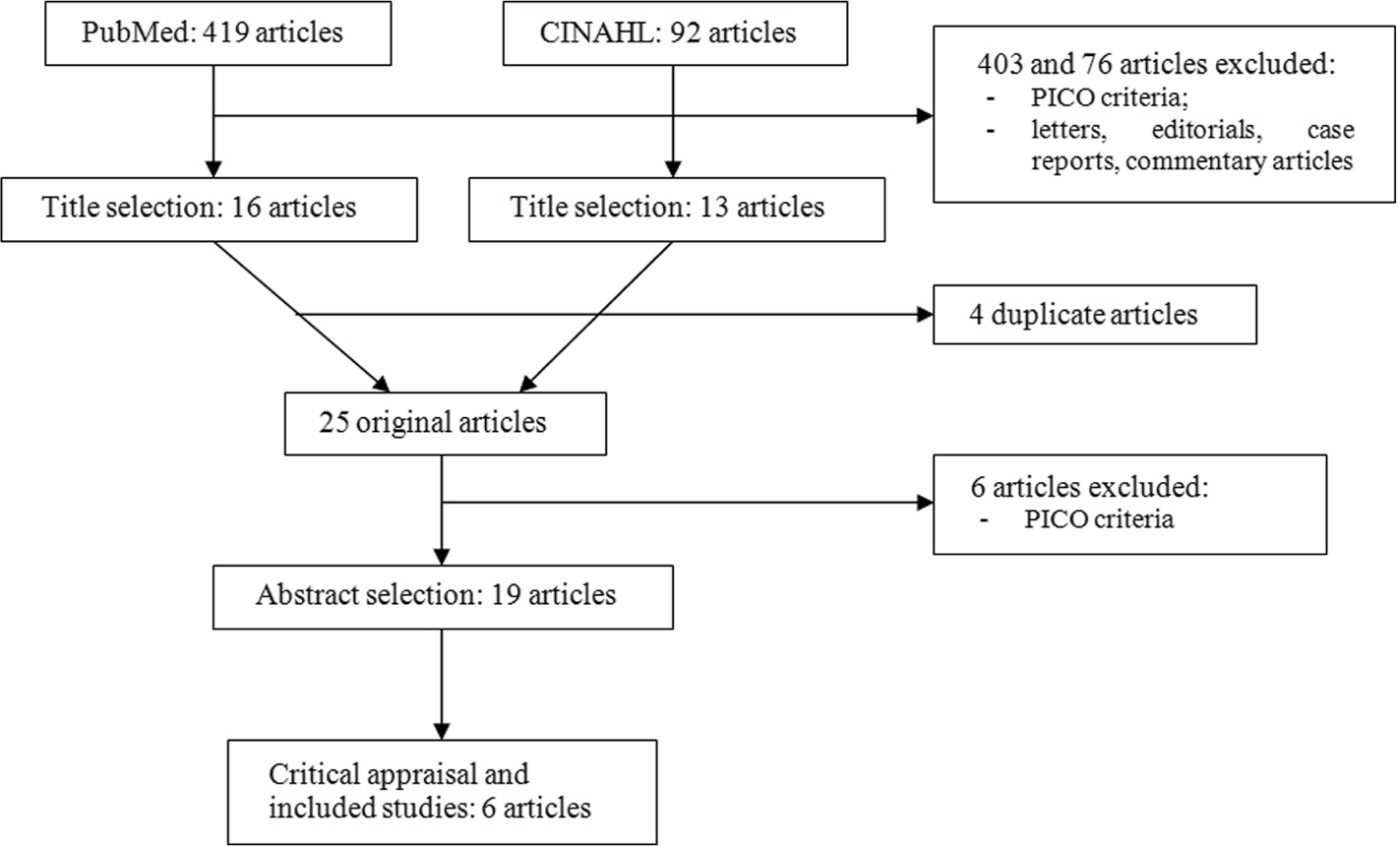
**Flow chart of the papers retrieved.** Legend: PICO: Patient/Population, Intervention, Comparison, Outcome, a method of putting together the better search strategy; CINAHL: Cumulative Index to Nursing and Allied Health Literature, a data bank.

Therefore, a second broader search was built on Pubmed and CINAHL, combining the following terms: (Dialysis OR Amyloidosis OR Myeloma OR Pain OR Fatigue) AND (Reiki OR (Healing touch) OR (Touch therapy) OR (Therapeutic touch) OR (Laying on of hands)). The search, limited to the last 5 years, on the account of the date of last updating of the Cochrane Review and to article in English, retrieved both a relevant Cochrane review and a series of 5 recent RCTs on Reiki and chronic pain (Table 2, Figure 1) [63-67]. The studies are highly heterogeneous, both in the Cochrane review (24 studies) and in the subsequent years (5 RCTs). Pain was assessed by various methods, with a visual analogue scale being the one most commonly used; control groups were different and the reasons for pain encompassed different diseases. Within these limits, the main results support a significant reduction of pain in patients undergoing touch therapies in general and Reiki in particular (Table 2). The overall quality of the review and of the selected RCTs was high (Table 2), in line with recent reports of a comparable quality of studies on CAMs and “mainstream Medicine” at least in the English language [34].

**Table 2:** Characteristics of selected articles

**Author, year**	**Study design**	**Participants**	**Measurements**	**Treatment**	**Comparison**	**Outcomes**	**Results**	**Side effects**	**CASP score**
**So, 2008**	Review	24 studies (1153 participants)	VAS, NRS, McGill Pain Index, SF-36, analgesic usage, MPAC, FACT	Touch therapies(TT): Reiki, Healing Touch, Therapeutic Touch	Sham placebo or ’no treatment’ control	Pain (acute or chronic)	Statistically significant reduction of pain with different treatment, especially with Reiki (95% CI: -1.16 to −0.50)	Not evaluated	7/10
**McCormack, 2009**	RCT	n=90 elderly patients with post-surgical pain: n=30 non-contact therapeutic touch, n=30 metronome treatment, n=30 no treatment	VAS, MPAC,TAS,HAT,pupil size	Reiki	Routine care, placebo	Post-operative pain	Statistically significant reduction of pain in the Reiki group, worsening of pain in the metronome group (p<0.01)	Not reported	7.5/10
**MacIntyre, 2008**	RCT, not blinded	n= 290 patients (mean age 64) undergoing first time elective coronary artery bypass surgery n=237 at the end of the study	MEDD, STAI	Healing Touch	Visitors and no intervention	Post-operative pain, anxiety, physical and mental status, length of stay	Significant reduction of hospital stay and anxiety. No significant reduction of pain	Not reported	7.5/10
**Frank, 2007**	RCT, patients, data collection staff and data analyst blinded	n= 82 females undergoing Stereotactic Core Breast Biopsy: n=42 intervention, n=40 placebo	VAS	Therapeutic Touch (TT)	Sham Reiki	Post-biopsy pain, lidocaine/ epinephrine dosage	Increase of pain in both groups, not statistically significant	Increase of pain in both groups	7.5/10
**Assefi, 2008**	RCT, patients, data collection staff and data analyst blinded	n=100 adults with fibromyalgia (23 real direct Reiki: 24= real distant Reiki, 23= sham direct Reiki, 23=sham distant Reiki)	VAS	Reiki	Sham Reiki	Pain, fatigue, sleep quality, well-being	Neither Reiki nor touch improve the symptoms of fibromyalgia in all groups	Not reported	7.5/10
**Aghabati 2010**	RCT	n= 90 patients with cancer and normal level of consciousness, age 15–65: n=30 TT, n=30 placebo, n=30 control	VAS, RFS	Therapeutic Touch (TT)	Mimic therapeutic touch and no intervention	Pain, fatigue	Statistically significant decrease in pain and fatigue in TT vs placebo or control (p=0.04)	Excess energy and anxiety in both groups	8/10

Legend: *RCT* Randomized Controlled Trial, *VAS* Visual Analogue Scale, *NRS* Numeric Rating Score, *SF-36* questionnaire for health-related quality of life, *MPAC* Memorial Pain Assessment Card, *FACT* Functional Assessment of Cancer Therapy, *TAS* Tellegen Absorption Scale, *HAT* Health Attribution Test, *MEDD* Morphine-Equivalent Dosage, *STAI* State Trait Anxiety Inventory, *RFS* Rhoten Fatigue Scale.

Nevertheless, statistical significance is not synonymous with clinical relevance: in the Cochrane review, the mean reduction of pain was less than one unit on a 0–10 scale, a limit probably not perceived by human beings, and in the 5 RCTs published after the review, Reiki was effective on pain in 2 studies and had no significant benefit in 3. Pain reduction was measured with a VAS scale in both articles and can be approximated to 1.5 cm (Table 2).

No study reported adverse events linked to the procedure.

Side effects were not specifically reported in the Cochrane review. Only one out of the 5 RCTs published after the Cochrane review reported specifically on side effects; they were described both in the Reiki and Sham Reiki groups. The main side effects were “excess energy” or anxiety (41%); 18% reported worsening of sleep or depression. The side effects were no different with Reiki or placebo [66]. One other study showed an increase of pain in groups of patients undergoing Stereotactic Core Breast Biopsy treated with Touch Therapies and with Sham Reiki [65]. This increase in pain-anxiety was presumably linked to the fact of “being studied”, suggesting that even the placebo effect may be two-faceted and that physicians should also control for the negative interferences of “sham” treatments.

### The EBM approach may not be sufficient to answer whether or not to facilitate Reiki in the dialysis ward without the application of an ethical framework

The overall picture deriving from the first steps of our analysis is thus of a widely used treatment of significant but limited efficacy, devoid of side effects, in no case inferior to placebo or the controls (Table 2).

While decisions on vital treatments, such as antibiotics or anti-neoplastic drugs, are mainly based on efficacy, decisions on chronic therapies, such as antihypertensives or on support therapies take into great account the expected side effects, leading some experts to conclude that the least effective treatment may occasionally be the best choice [16,68-70].

The shift from efficacy to tolerance has important philosophical implications.

The analysis according to the four main principles of principlist ethics may be a useful pragmatic guide for analysis [38-42]. The principles may be defined and contextualized as follows: beneficence - actions intended to benefit the patient; this was considered equivalent to therapeutic efficacy. Non-maleficence- actions intended not to harm or bring harm to the patient; this was considered equivalent to side effects. Justice - defined as being fair or just to the wider community in terms of the consequences of an action; this was considered to include the costs of therapy and the eventual integration with other treatments. Autonomy - respect for individuals and their ability to make decisions with regard to their own health and future; this was considered a reason to favour all non-maleficent therapies when chosen by the patient. The principle of beneficence supports a limited positive effect of Reiki, hardly perceivable in terms of pain decrease, thus questioning the opportunity of the integration of this therapy in the dialysis ward. However, the lack of relevant side effects (non-maleficence), the potential integration with other therapies and the negligible costs, at least in settings where Reiki is offered by non-profit volunteer associations (justice), together with the desire of the patient to “do something” for his pain (autonomy), on the contrary clearly support the choice of integrating Reiki into the patient’s therapies.

These considerations are in line with the suggestions of the Society for Integrative Oncology in the case of treatments with limited efficacy but without relevant side effects: “tolerate, encourage caution, closely monitor effectiveness” [71].

## Conclusions

The growing diffusion of CAMs in chronic diseases will increasingly confront the Nephrologist with the problem of integrating CAMs into Renal Replacement Therapies; this topic is a novelty for most Nephrologists and there is a need to acquire problem-solving tools.EBM offers an analytical pathway that is very interesting in the case of new diseases or non-codified therapies and is particularly suitable to the study of CAMs. The integration of an ethics-based discussion may offer interesting tools to systematically face such issues.

In the case of Reiki, the results of a systematic review, supplemented by a further updating, demonstrate a statistically significant but clinically barely relevant benefit. The use of Reiki should therefore be probably discouraged if only efficacy is considered, but chosen if the emphasis is on “non-maleficium” or the patient’s autonomy; the issue of justice modulates the choice according to the burden of overall costs, and the availability of the treatment in the different settings.

The additional need of an ethical discussion based on sound evidence-based results to tackle new problems in our “old” context is in line with the approaches suggested in a different field by the Society for Integrative Oncology in the case of treatments with limited efficacy but without relevant side effects: “tolerate, encourage caution, closely monitor effectiveness” [71].

## Summary

The present debate, integrating evidence based approaches and ethical framework, tries to balance the pros and cons of the systematic introduction of such approaches in the dialysis ward. In spite of the wide use of CAMs in the general population, few studies deal with the pros and cons of an integration of mainstream medicine and CAMs in dialysis patients; one paper only regarded the use of Reiki and related techniques. According to the EBM approach, Reiki allows a statistically significant but very low-grade pain reduction without specific side effects. However, the ethical discussion leads to a permissive-positive attitude.

This paper suggests that physicians may wish to consider efficacy but also side effects, contextualization (availability and costs) and patient’s requests, according also to the suggestions of the Society for Integrative Oncology (tolerate, control efficacy and side effects).

## Competing interests

The authors declare that they have no competing interest.

## Authors’ contributions

GBP and MF conceived and wrote the manuscript. NC provided the historical references and perspective. RC, IM, EB, EB, AC, RD, LR, ES, AS AT, working in group in the EBM course of the san Luigi Medical School of Torino, Italy, performed the Internet searches, retrieved the evidence, draw the tables, drafted different parts of the manuscript, and reviewed the text. All authors read and approved the final manuscript.

## Pre-publication history

The pre-publication history for this paper can be accessed here:

http://www.biomedcentral.com/1471-2369/14/129/prepub
